# Integrated Analysis of Neuroendocrine and Neurotransmission Pathways Following Developmental Atrazine Exposure in Zebrafish

**DOI:** 10.3390/ijms252313066

**Published:** 2024-12-05

**Authors:** Sydney C. Stradtman, Jenna N. Swihart, Kaylin Moore, Isabelle N. Akoro, Janiel K. Ahkin Chin Tai, Wagner Antonio Tamagno, Jennifer L. Freeman

**Affiliations:** School of Health Sciences, Purdue University, West Lafayette, IN 47907, USA; sstradtm@purdue.edu (S.C.S.);

**Keywords:** atrazine, dopamine, estradiol, hypothalamus, kisspeptin, zebrafish

## Abstract

Atrazine is an endocrine-disrupting herbicide, with exposure impacting adverse outcomes along multiple endocrine pathways. This study investigated the neuroendocrine system as the central target of atrazine toxicity, examining effects of early developmental exposures on neurohormones and genes associated with kisspeptin, hypothalamic, pituitary, and dopamine systems. Zebrafish were exposed to 0, 0.3, 3, or 30 ppb (µg/L) atrazine during two developmental time windows. For neurohormone assessments, exposure was ceased at the end of embryogenesis (72 h post-fertilization, hpf) and analyzed immediately or grown to 0.5, 2, or 2.5 years post-fertilization (ypf). Gene expression was measured immediately after 1–72 hpf or 72–120 hpf exposure. Estradiol decreased in the 0.3 and 30 ppb groups in 0.5 ypf female brains, while dopamine decreased in the same treatment groups at 72 hpf. Increases were also observed in 2.5 ypf female brains (3 ppb) for estradiol and in 2 ypf female and male brains (3 and 30 ppb) for dopamine. Gene expression alterations occurred for the follicle-stimulating hormone (*fsh*) at 72 hpf and the growth hormone (*gh1*) at 72 and 120 hpf. Overall, results indicated that developmental atrazine exposure has immediate and long-term sex-specific effects on neurohormonal systems.

## 1. Introduction

Atrazine [6-chloro-N-ethyl-N-(1-methylethyl)-1,3,5-triazine-2,4-diamine] (ATZ) is a triazine herbicide that is widely used to control the growth of broadleaf and grassy weeds. Atrazine is the second-most-used herbicide in the United States, although it has been banned for use in the European Union since 2003, with residual atrazine and metabolites left in the environment and drinking water not allowed to exceed 0.1 µg/L (parts per billion, ppb) due to its endocrine-disrupting effects in humans and multiple biological models [1,2]. Atrazine is a known endocrine-disrupting chemical (EDC) and is therefore regulated in the United States by the Environmental Protection Agency (EPA). The greatest risk of exposure to the general population is through contaminated drinking water consumption. The maximum contaminant level (MCL) that the US EPA has set for atrazine in drinking water is 3 ppb. Oftentimes, this level can be exceeded during peak crop seasons, and there is a possibility of contaminating ground and surface drinking water in close proximity to where atrazine application occurs [3,4,5,6]. Importantly, this study worked to predict atrazine concentrations in waterbodies through the inclusion of a multitude of variables that can influence contamination risk.

The US EPA MCL is based solely on the disruption that atrazine has on the luteinizing hormone surge in the neuroendocrine control of the reproductive system, although the literature has shown a multitude of adverse effects along the endocrine axes and other systems in the body at concentrations surrounding 3 ppb [2,7,8,9,10,11,12]. The adverse effects reported on atrazine in the literature are a public health concern due to the persistence of atrazine in the environment after use, causing continuous leaching into drinking water sources, leading to longer exposure periods.

The atrazine literature is well developed in reporting the adverse effects on development and reproductive system outcomes in different species. In humans, epidemiological studies associated atrazine with negative impacts on development and reproduction such as small-for-gestational-age births, an increase in the chance of preterm birth, birth defects, menstrual cycle irregularities in women, and reduced semen quality in men [3,4,13,14,15,16,17,18]. Toxicological studies support these findings, describing reduced gonadotropin-releasing hormone (GnRH) pulse frequency, luteinizing hormone (LH) and follicle-stimulating hormone (FSH) surges, and serum and intratesticular testosterone in rats [9,10,19,20,21,22]. Studies with zebrafish report increased progesterone and follicular atresia in the ovaries of adult females exposed to atrazine only during embryogenesis [23], along with similar effects in multiple other animal species spanning from amphibians to marsupials [24,25]. These studies report a vast array of adverse effects observed across the neuroendocrine axes that lead to reproductive system perturbations. Although it is acknowledged by the US EPA and other regulatory agencies that atrazine in adult females disrupts the luteinizing hormone surge in the neuroendocrine control of the reproductive system, an upstream specific target of atrazine toxicity has yet to be identified to elucidate the variety of adverse impacts reported in both sexes along the reproductive system.

Furthermore, in addition to atrazine influencing the reproductive system, there are several studies that report the effects it has on neurotransmission, behavior, and behavior disorders. For example, in zebrafish, low environmentally relevant concentrations of atrazine during development significantly altered larval behavior, causing hyperactivity at 0.3 ppb and hypoactivity at 30 ppb [8]. Further, reports on rats and mice show decreased dopamine levels after developmental or adult exposures [26,27,28,29]. Although rodent studies provide insight into the perturbations caused by atrazine exposure on the dopaminergic system, the exposures used were high in relation to the environmentally relevant concentrations found in drinking water. A gap exists in the literature in assessing the dopaminergic system following an environmentally relevant exposure to atrazine. Moreover, while dopamine is mostly considered a neurotransmitter, it also plays important roles in the neuroendocrine system, specifically in the hypothalamic–pituitary–adrenal axis and the hypothalamic–pituitary–gonadal axis [30]. As such, it is imperative to consider dopamine as both a neurotransmitter and a neurohormone when looking at adverse effects associated with atrazine exposures.

Given that the atrazine literature describes adverse outcomes on both the neuroendocrine axes and neurotransmission, yet no connection to a common target of atrazine has been identified, it is important to examine the outcomes along these pathways alongside each other. Examining these pathways simultaneously, assuming they are connected by a central target of atrazine, is an approach that can provide the insight needed to establish a common target of atrazine toxicity and a clearly defined mechanism. A lack of comparisons and analyses of connections between the wide array of adverse effects and alterations in multiple different biological systems and pathways linked to atrazine exposure is the main reason why a clear, defined mechanism of atrazine toxicity has yet to be elucidated. Although it is evident, based on a plethora of studies, that atrazine has both immediate and prolonged adverse effects on multiple biological pathways, especially along the endocrine axes and neurotransmission, there is a gap in the literature that needs to be addressed regarding the reason these outcomes are observed and a possible connection between all of the adverse effects reported. It is especially important to understand atrazine toxicity due to the ability of the herbicide to cross the placental and blood–brain barriers [31]. The most common route of exposure is contaminated drinking water; this puts developing fetuses particularly at risk for alterations in the neuroendocrine and neurotransmission systems before birth that can have a lifelong impact. The stages of neurogenesis and the structures and pathways in the brain are established at specific timepoints throughout development, so there are varying adverse effects of atrazine dependent on the timing of exposure, which is important to take into account as well [32]. Therefore, it is important to evaluate the effect that environmentally relevant exposures to atrazine during multiple key developmental windows can have immediately and later in life while trying to understand the central target of atrazine toxicity during these exposure windows.

**Figure 1:**
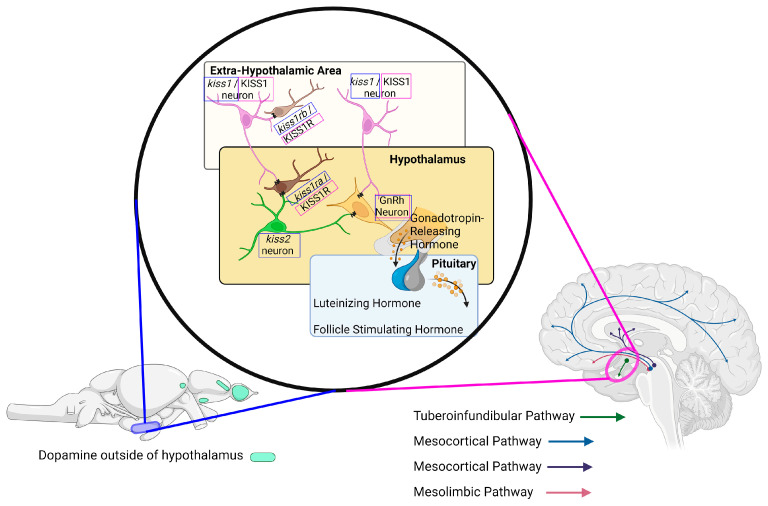
Hypothalamus outlined in both the zebrafish brain (**left**) and the human brain (**right**), which shows the kisspeptin pathway and innervations of neurons. The mammalian kisspeptin signaling system is conserved in zebrafish, with the exception of zebrafish having two kisspeptin genes (*kiss1* and *kiss2*) and two receptors (*kiss1ra* and *kiss1rb*). The zebrafish-specific genes are contained within a pink box and the human equivalents are contained within a blue box. The kisspeptin system is a reproductive regulator as well as a dopamine regulator, which is highlighted by showing the proximity of the dopaminergic pathways in the human brain. Dopamine is found within the hypothalamus in the zebrafish brain (blue) and in other locations—as in the human brain—which are denoted in green. This image shows the conserved neuroendocrine system and key components in regard to the kisspeptin system and signaling amongst the human brain and the zebrafish brain. It also outlines the similarities in the dopaminergic systems between human and zebrafish brains to show the viability of the zebrafish model to study atrazine neuroendocrine toxicity.

The kisspeptin signaling system is an upstream regulator of both GnRH hormone surges and reproduction as well as dopamine and serotonin transmission [33,34]. A study on female rats recently reported the adverse effects atrazine has on the kisspeptin signaling system, describing a decrease in *Kiss1* mRNA expression levels [35]. Based on the reported adverse outcomes in the literature following atrazine exposure, further investigation into the kisspeptin peptide signaling system is essential in elucidating the mechanism of atrazine toxicity due to the connection between the pathways that kisspeptin regulates and the described effects of atrazine exposure (Figure 1).

Examining neurotransmission and neuroendocrine outcomes and considering the role of dopamine as a neurohormone can be an impactful approach leading toward the central target of atrazine toxicity. Therefore, it was hypothesized that an environmentally relevant exposure to atrazine during development would lead to downstream changes of the reported outcomes in the literature, examining dopamine and estradiol concentrations, and the origin of these alterations could be identified at the gene level. To address this research question, the zebrafish was used as a biological model for human health. The zebrafish is a well-established and accepted whole animal model for investigating developmental toxicity, neurotoxicity, and endocrinology [36,37,38]; translation to human health, including past atrazine studies detecting the same major metabolites [8]; and similar perturbed molecular pathways and functional outcomes along the endocrine axes [11,12,23,39,40,41]. Also, for this study, the route of exposure of atrazine to the developing zebrafish embryos mimicked in utero exposures that occur during human development, further solidifying the viability of the model. Moreover, the intersection of neurotransmission and neuroendocrine regulation also occurs in zebrafish, with similarities and differences defined (Figure 1) [42,43], making them an acceptable model for this study.

## 2. Results

### 2.1. Embryonic Atrazine Exposure Impacts on Estradiol Concentrations

**Figure 2:**
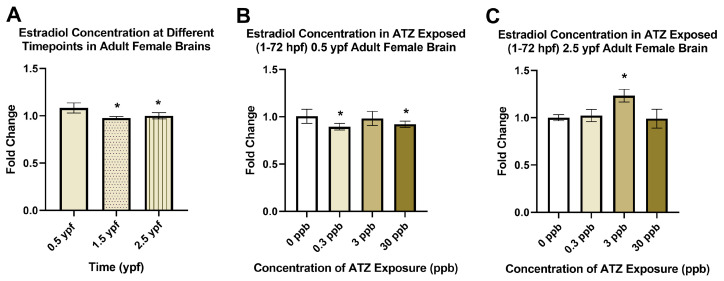
Control AB wild-type adult brains were compared at 0.5 ypf, 1.5 ypf, and 2.5 ypf (**A**). At 1.5 and 2.5 ypf, estradiol concentrations were found to be significantly lower than at the 0.5 ypf timepoint (*p* < 0.05, n = 3). Estradiol concentration was then measured after 0, 0.3, 3, or 30 ppb exposure to atrazine (ATZ) between 1 and 72 hpf. Adult female brains were collected at 0.5 ypf (**B**) and 2.5 ypf (**C**). Significant decreases were observed at 0.3 ppb and 30 ppb in 0.5 ypf adult female brains, and a significant increase was observed at 3 ppb in 2.5 ypf adult female brains due to embryonic atrazine exposure (*p* < 0.05, n = 3–8). Error bars indicate standard deviation. * *p* < 0.05.

A time course study was first completed to analyze estradiol concentrations in the brains of adult female zebrafish at 0.5, 1.5, and 2.5 ypf, reflecting different stages of reproductive activity (i.e., 0.5 ypf: mid-life prime reproductive age; 1.5 ypf: aged adult at or near reproductive cessation; 2.5 ypf: extreme-aged adult in reproductive cessation). Results indicated that estradiol concentrations were significantly lower at the later life stages, 1.5 and 2.5 ypf, when compared to the prime-breeding-age zebrafish at 0.5 ypf (Figure 2A).

Estradiol concentrations were then examined following embryonic atrazine exposure (1–72 hpf) in whole eleuthero embryos at 72 hpf and in adult zebrafish brains at 0.5 and 2.5 ypf (i.e., prime reproductive and reproductive cessation stages, respectively). Absorbance readings measured were inversely related to the concentration of estradiol in the samples. Foldchange calculations were completed, comparing exposure groups to the negative control (0 ppb). At 0.5 ypf, female zebrafish brains exposed to 0.3 or 30 ppb atrazine during embryogenesis had decreased estradiol (Figure 2B), while, at 2.5 ypf, female zebrafish brains showed a significant increase in the 3 ppb treatment group (Figure 2C). No significant alterations in estradiol were observed at 72 hpf nor in adult male brains at 0.5 or 2.5 ypf (Supplementary Figure S2).

### 2.2. Embryonic Atrazine Exposure Impacts on Dopamine Concentrations

**Figure 3:**
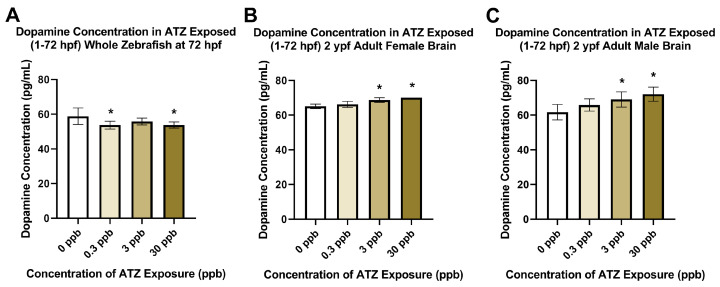
Dopamine concentration was measured after 0, 0.3, 3, or 30 ppb exposure to atrazine (ATZ) between 1 and 72 hpf. Populations of eleuthero embryos were collected at 72 hpf (**A**), adult female brains were collected at 2 ypf (**B**), and adult male brains were collected at 2 ypf (**C**). Significant decreases in dopamine were observed in atrazine-exposed larvae at 0.3 and 30 ppb and significant increases were observed in adult female and male brains in the 3 and 30 ppb treatment groups (*p* < 0.05, n = 4–6). Error bars indicate standard deviation. * *p* < 0.05.

Dopamine concentrations measured in both whole eleuthero embryos and adult brains with embryonic atrazine exposure showed significant alterations in different exposure groups. At 72 hpf, zebrafish exposed to 0.3 or 30 ppb atrazine had significant decreases in dopamine concentrations (Figure 3A). Adult female and male brains at 2 ypf showed the same response, with significantly higher concentrations of dopamine for those in the 3 and 30 ppb treatment groups (Figure 3B,C).

### 2.3. Gene Expression Alterations in Neuroendocrine Pathways

**Figure 4:**
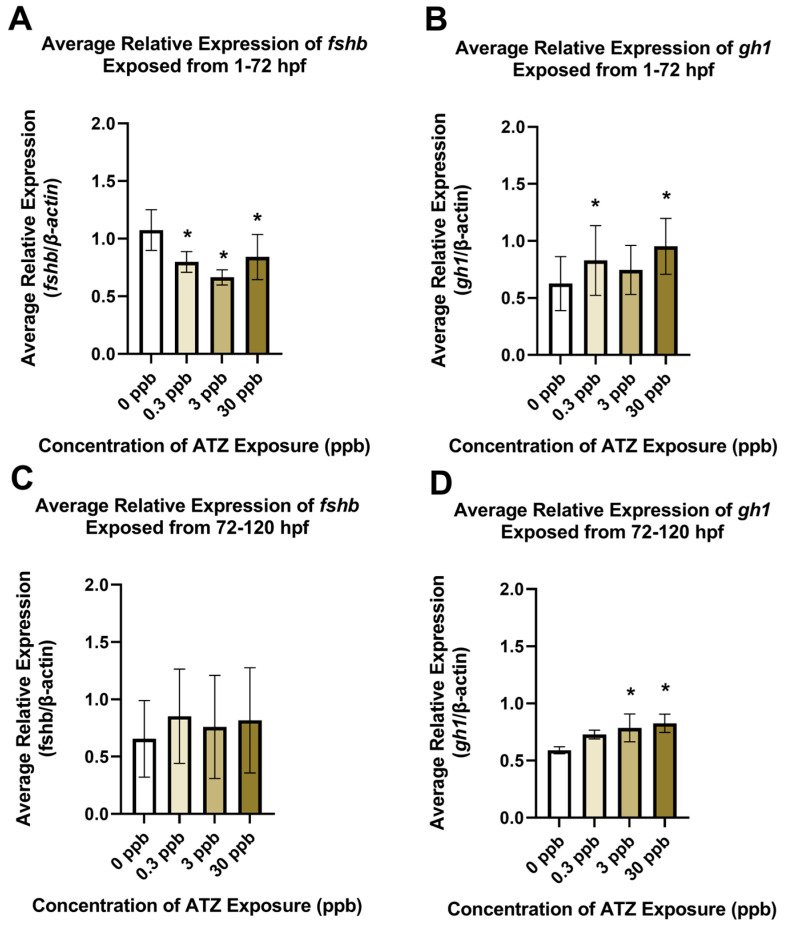
Gene expressions of *fshb* (**A**,**C**) and gh1 (**B**,**D**) were measured after 0, 0.3, 3, or 30 ppb exposure to atrazine (ATZ) between 1 and 72 hpf and 72 and 120 hpf. Significant decreases in gene expression in *fshb* at 72 hpf were observed in all treatment groups (**A**) (*p* < 0.05, n = 4). No significant changes in gene expression were detected in *fshb* at 120 hpf (**C**) (*p* > 0.05, n = 7). Significant increases in *gh1* gene expression were observed at 72 hpf (**B**) in the 0.3 and 30 ppb treatment groups (*p* < 0.05, n = 6), while significant increases in *gh1* were also observed at 120 hpf (**D**) in the 3 and 30 ppb treatment groups (*p* < 0.05, n = 4). Error bars indicate standard deviation. * *p* < 0.05.

A targeted assessment of multiple genes associated with hormones known to play a role in pathways along the neuroendocrine axis was conducted following embryonic atrazine exposure (1–72 hpf). Significant alterations occurred in *fshb* and *gh1*. A significant decrease in *fshb* gene expression was observed at 0.3, 3, and 30 ppb, while a significant increase in *gh1* gene expression was observed at 0.3 and 30 ppb (Figure 4A,B). No other significant alterations were detected in genes examined at this timepoint (Supplementary Figures S3–S5 and S6A–D).

Next, a subset of the genes (*fshb*, *gh1*, *gnrh*, *lhb*, *kiss1*, *kiss2*, *kiss1ra*, *kiss1rb*) was then examined following larval atrazine exposure (72–120 hpf). Although significant alterations were observed in *fshb* gene expression at 72 hpf, there were no significant alterations observed after 72–120 hpf exposure (Figure 4C). However, the gene expression of *gh1* did exhibit significant increases in gene expression after 72–120 hpf atrazine exposure as it did at 72 hpf. The later exposure period affected the 30 ppb exposure group, as it did in the earlier exposure group and the 3 ppb exposure group (Figure 4D). No other significant alterations were observed following larvae atrazine exposure in the other molecular targets examined (Supplementary Figures S6E–H and S7).

## 3. Discussion

The effects that atrazine has on the neuroendocrine system as reported for other biological models is supported by the results observed in this study. A novel approach was adopted, examining dopamine and estrogen as neurohormones and analyzing both neurotransmission pathways and neuroendocrine pathways alongside each other to explore a potential common upstream target of atrazine through multiple analyses following embryonic atrazine exposure. We first examined estradiol throughout the lifetime of male and female zebrafish in the brain. First, we conducted a time course study in adult female zebrafish to investigate the abundance of estradiol at the beginning of prime breeding age (0.5 ypf), at the end of prime breeding age (1.5 ypf), and at geriatric age past the prime breeding period (2.5 ypf). The findings aligned with the literature, confirming that the highest concentration of estradiol in the female zebrafish brain is during prime breeding age at 0.5 ypf when compared to the other timepoints. Specifically, the results follow a similar pattern observed during the life cycle of human females, where estradiol levels are highest during reproductive years and decline following the final menstrual period [44]. Several studies evaluated alterations in estradiol with atrazine exposure in multiple species, but all measured concentrations in the blood or gonads [23,45,46,47]. Estradiol is also a neurohormone, with estrogenic receptors present in specific neuronal populations in the brain. In the current study, the goal was to specifically focus on estradiol as a neurohormone, given the hypothesis that downstream effects can be traced to neuroendocrine alterations.

Following this analysis, embryonically exposed adult female brains at 0.5 ypf demonstrated a decrease in estradiol concentrations with 0.3 and 30 ppb atrazine. This finding aligns with the literature in regard to decreases in GnRH and LH and FSH surges causing a decrease in estrogen synthesis. This decrease did not demonstrate a concentration-dependent response, suggesting that compensatory mechanisms may be at play at around the 3 ppb concentration that are then overcome again by the higher 30 ppb concentration. These findings align with the 72 hpf dopamine ELISA results as well as behavior studies, which are further discussed below, that also lacked a concentration-dependent response, This was found to be a common finding amongst the atrazine literature with low dose exposures.

At 2.5 ypf, an increase in estradiol was observed at 3 ppb, which further confirms the ability of atrazine to dysregulate hormone signaling and endocrine axes at late stages in life with exposure much earlier in life. In postmenopausal women, GnRH, LH, and FSH surges tend to decrease with age, which is partially compensated for by a baseline GnRH secretion [48]. The results of our time course study of estradiol demonstrated this, with an overall decrease in estradiol from 0.5 ypf to 2.5 ypf. The changes in these biological pathways as the female zebrafish aged, leading to less of a drastic fluctuation in these key hormones that caused a decrease in estradiol in the older control fish, can help to explain why there was an increase in estradiol in the older fish exposed to atrazine. While several studies show decreases in estrogen following atrazine exposure, there are also additional studies reporting that atrazine increases estrogen synthesis [25]. In our study, the time in which estradiol levels were measured appeared to play a role in how estrogen levels were affected, which may be connected to the biological changes in other hormones along pathways that affect estrogen synthesis as female zebrafish age. Therefore, the absence of the surges that control the synthesis and secretion of estrogen in older fish may allow for the effects of atrazine on this biological pathway to increase the concentrations of estradiol in the brain. This observation is due to the age of the fish and the hormone levels that are at play along these pathways at different timepoints throughout the lifetime of the fish. The perturbations in estradiol concentration that were present in the female brains following atrazine exposure align with previous studies by our group on adult females that show increases in upstream targets, such as gene expression changes of LH, and progesterone, which acts as a negative inhibitor of the neuroendocrine pathway, which triggers the release of estrogen [23,40,49]. The changes in estradiol concentration also coincide with adverse functional outcomes along the reproductive axes reported in our earlier study, such as decreases in spawning and an increase in follicular atresia in zebrafish exposed to 30 ppb atrazine [23].

Although no significant changes in estradiol concentrations were observed amongst eleuthero embryos at 72 hpf or male brains at 0.5 and 2.5 ypf, these findings align biologically with the timepoint and the sex, respectively. At the end of embryogenesis at 72 hpf, both estradiol and testosterone were only beginning to be detected [36]. In zebrafish, sex differentiation initiates at 21–23 days post-fertilization (dpf), with sexual maturity occurring at around 3 months post-fertilization (mpf) [50]. Therefore, this initiation timepoint of androgen production at 72 hpf is likely to make this a less sensitive timepoint for hormone alterations. As for adult males, the primary, and most abundant, androgen in male zebrafish is testosterone [37]. Therefore, similar to the eleuthero embryos, the production of estradiol in the adult male zebrafish was not as prominent and likely not as sensitive to atrazine impacts. In addition, the lack of alterations in brain estradiol in the males aligns with our previous findings of equal sex ratios and no evidence of feminization in adult male zebrafish with embryonic atrazine exposure [6,23].

As mentioned, atrazine is not only known for its disruption of the neuroendocrine system but also for the adverse effects it has on neurotransmission and dopaminergic systems. We analyzed dopamine concentrations in exposed eleuthero embryos and adult brains to examine any alterations that align with the literature. Embryonically exposed eleuthero embryos had significantly lower concentrations of dopamine at 0.3 and 30 ppb, which aligns with many of the rodent studies that also report decreases in dopamine following exposure. As mentioned above, the lack of a concentration-dependent response may be due to compensatory actions via feedback loops that occur around 3 ppb that are overcome as the concentration increases to 30 ppb. With environmentally relevant low concentrations of atrazine coupled with developmental exposure, the compensatory mechanisms may activate opposing pathways at very specific concentrations in which the adverse response is overcome. As such, those same opposing pathways cannot be overcome at a higher concentration, resulting in the lack of a concentration-dependent response being observed. Overall, the chemical exposure within the body could reach a threshold that determines the activation or deactivation of specific pathways, such as those seen in tightly regulated systems including the endocrine systems in the body. These findings are consistent with previous studies by our group that report significant changes typically found in 0.3 and 30 ppb exposure groups in larval visual–motor response and acoustic startle behavior assays [7]. Alternatively, in the 2 ypf male and female brains, a significant increase in dopamine concentration occurred at 3 and 30 ppb. Many studies using rodents and cells to study atrazine’s effects on dopamine reported decreased intracellular dopamine in different parts of rat brains [26,28,37,51,52,53]. Our first timepoint analyzed (72 hpf) aligns with decreased dopamine found in previous rodent and cell studies, while our 2 ypf timepoint results do not align with these findings. However, one study on rats reported an increase in dopamine levels in the hippocampus at 10 mg/kg (their low-dose exposure) but not at 100 mg/kg (their high-dose exposure) [54]. The concentrations of atrazine used in our studies tend to be much lower than in many other rodent studies, which can explain why an increase was detected in the zebrafish at 2 ypf using environmentally relevant concentrations. Other factors that can explain this discordance are differences in exposure period, exposure route, time of analysis, and the brain tissue analyzed, especially given the advanced age of the fish. Previous studies by our group and others have identified gene targets associated with movement disorders that were modified in male zebrafish as well as disturbances in locomotion assessed in a behavior assay using a range of 0.3–300 ppb atrazine exposure treatment that are also linked to alterations in the dopaminergic system [11,39]. In these earlier studies by our group using the same atrazine concentrations and embryonic exposure period, larval and adult male zebrafish experienced hypoactivity with embryonic atrazine exposure [11,12]. Specifically, the adult males spent less time moving and had a decrease in total distance moved and velocity. It is important to note that dysregulation in dopaminergic signaling and overall synthesis can cause many neurological disorders, including schizophrenia and addiction when dopamine increases and Parkinson’s disease and Attention Deficit Hyperactivity Disorder (ADHD) when dopamine is decreased [55]. These findings should be further investigated in regard to the relationship between atrazine exposure and neurological disorders such as the disorders listed. Overall, the literature and this study support disruption of the dopaminergic system that is directly related to atrazine exposure, but further studies are needed to address these alterations observed.

Following observations of significant alterations in estradiol and dopamine concentrations from an embryonic atrazine exposure, gene targets associated with the neuroendocrine axes and dopaminergic systems were assessed to attempt to elucidate upstream target(s) to explain the perturbations. Two different exposure periods were assessed to examine adverse effects that align with the developmental processes that occur in these different timepoints (i.e., embryogenesis and larval). The first exposure period assessed (1–72 hpf) showed significant decreases in *fshb* gene expression at 0.3, 3, and 30 ppb, which aligns with the literature that reports a decrease in FSH [10]. A significant increase in *gh1* at 0.3 and 30 ppb was also observed at this timepoint. These results do not display a concentration-dependent response, much like the results from the ELISA assays discussed above. However, data from lower atrazine exposure levels commonly do not display concentration- or dose-dependent responses due to the complex nature of potential compensatory mechanisms and thresholds for specific exposure concentrations, as mentioned previously. Significant increases were also observed at 3 and 30 ppb after an exposure period from 72–120 hpf. This coincides with previous work in our lab using the same atrazine concentrations and exposure period that reported increases in F0-atrazine-exposed larval head length, head-to-body ratio, and brain size and increases in F1-azatrine-exposed head length and the ratio of head length to total length [7,12,40].

Further investigations into the kisspeptin system were completed at the molecular level due to significant findings of *fshb* at the gene level and estradiol and dopamine at the hormone level, all of which are downstream signaling hormones regulated by kisspeptin. In mammals, one kisspeptin gene exists, *kiss1*, and takes on the function of regulating both reproductive pathways and dopamine and serotonin transmission [56,57]. However, zebrafish express two different kisspeptin genes, *kiss1* and *kiss2*. The regulation of dopamine and serotonin is described to be the responsibility of *kiss1*, while the regulation of reproduction has been described to be the responsibility of *kiss2*. Zebrafish also have two different genes expressing different kisspeptin receptors, *kiss1ra* and *kiss1rb*, with *kiss1ra* having an affinity to bind with *kiss1* and *kiss2* and *kiss1rb* having the affinity to bind with *kiss1* [43]. Therefore, when beginning to examine the kisspeptin system in zebrafish following atrazine exposure, both kisspeptin genes and their receptors were investigated to cover the range of neurotransmission and neuroendocrine adverse outcomes that were discovered. No significant alterations in gene expression at any of the environmentally relevant concentrations of atrazine exposure were found at 1–72 hpf or 72–120 hpf for any of the kisspeptin-related gene targets. The kisspeptin system is not well established until the time of puberty and sexual maturity [58]. As such, the timepoints addressed in this study may have been too early to detect changes in kisspeptin gene expression at early stages of development. Though changes were not found, the results of this study show that atrazine’s target is upstream of dopamine and estradiol, which may not be seen at the gene level or may be reflected at a different analysis timepoint, given gene expression dynamics during development. Further investigation into different aspects of the kisspeptin signaling system is necessary to elucidate a mechanism of atrazine toxicity that coincides with the wide array of adverse outcomes across multiple systems and pathways that can connect back to the regulator kisspeptin.

## 4. Materials and Methods

### 4.1. Chemical Preparation and Concentration Confirmation

A 10-parts-per-million (ppm: mg/L) atrazine stock solution made with 98.1% purity atrazine (CAS, 1912-24-9; Chem Service, West Chester, PA, USA) was prepared as described in [40,41]. Filtered aquaria water was used to dilute the stock into the exposure solutions of 0.3, 3, and 30 parts per billion (ppb; µg/L). A negative control of 0 ppb consisted of filtered aquaria water. An enzyme-linked immunosorbent assay (ELISA) kit (Abraxis, Gold Standard Diagnostics, Horsham, PA, USA) approved by the US EPA was used to confirm the concentrations of the exposure treatments [59]. Within each treatment group, measured atrazine concentrations ranged as follows: 0 ppb: below detection limit; 0.3 ppb: 0.27–0.35 ppb (average: 0.31 ppb); 3 ppb: 2.9–3.6 ppb (average 3.2 ppb); and 30 ppb: 28–34 ppb (average: 31.1 ppb).

### 4.2. Zebrafish Husbandry and Atrazine Dosing Confirmation

Zebrafish (*Danio rerio* AB wild-type strain) were housed in a LabREED Zebrafish System (Iwaki Aquatic, Holliston, MA, USA) on a 14:10 light–dark cycle and monitored daily to maintain a temperature of 28 °C, a pH range of 7.0–7.3, and a conductivity range of 470–550 μS. Fish were fed twice daily with a mixture of brine shrimp (*Artemia franciscana*) (Artemia International LLC, Fairview, TX, USA), Zeigler adult zebrafish food (Zeigler Bros Inc., Gardners, PA, USA), and Golden Pearls (500–800 μm) (Artemia International LLC, Fairview, TX, USA). Adult fish were bred and embryos were collected from breeding tanks at the 4–8 cell stage [1 h post-fertilization (hpf)] [60,61]. Embryos were rinsed and randomly sorted into Petri dishes in groups of 50 and assigned a treatment and exposure period (Supplementary Figure S1). Two different exposure periods were used. For the first exposure period, embryos were submerged in a 20 mL solution of 0.3, 3, or 30 ppb (μg/L) atrazine and exposed at 28 °C from 1 to 72 hpf (end of embryogenesis). Eleuthero embryos were rinsed with filtered aquaria water to cease exposure and either collected at 72 hpf or raised with general husbandry conditions to 0.5, 2, or 2.5 years post-fertilization (ypf). The second exposure period consisted of development at 28 °C from 1 to 72 hpf in 20 mL of filtered aquaria water only. At 72 hpf, filtered aquaria water was replaced with 20 mL of either 0, 0.3, 3, or 30 ppb atrazine for exposure from 72 to 120 hpf. At 120 hpf, larvae were rinsed with filtered aquaria water and collected. Two exposure periods were analyzed because of the different structures and functioning capacities of the central nervous and neuroendocrine systems at these stages of development. Dopamine neurons and receptors were first observed before 24 hpf, while kisspeptin gene expression was measured throughout embryogenesis and larval development [38,42]. However, the blood–brain barrier develops significant function at 72 hpf and beyond [62,63]. Collection of eleuthero embryos/larvae occurred at timepoints prior to sex differentiation. Confirmation of sex was completed at adult stages by examination of the gonads. Investigators were blind to treatment concentrations for outcome analysis. All animal protocols were approved and performed in accordance with Purdue University’s Institutional Animal Care and Use Committee guidelines.

### 4.3. Estradiol Levels in Whole Eleuthero Embryos and Adult Brains

First, given known fluctuations of estradiol in mammalian females and other species during the lifetime, brains from adult female zebrafish at 0.5, 1.5, and 2.5 ypf were extracted after euthanizing fish in tricaine for the time course evaluation of baseline estradiol concentrations throughout the adulthood of female zebrafish. Next, zebrafish exposed to atrazine during embryogenesis (1–72 hpf) were collected immediately at the end of the exposure period (72 hpf) in groups of 45 and homogenized manually using a pestle and microfuge tube in 200 µL of Estradiol ELISA Buffer (Estradiol ELISA Kit, Item No. 501890; Cayman Chemical,, Ann Arbor, MI, USA). In addition, the brains of male and female adult zebrafish aged 0.5 or 2.5 ypf with embryonic atrazine exposure (1–72 hpf) were extracted. Wet fish mass and brain mass were recorded. Brain tissue was then flash-frozen in screw-cap tubes in liquid nitrogen and stored at −80 °C. At the time of assessment, brains were homogenized manually using a pestle and microfuge tube and put through three freeze–thaw cycles at −20 °C, followed by centrifugation at 7500× *g* for 5 min. The supernatant was then removed and the total protein concentration was measured using NanoDrop^TM^ One (Thermo Fisher Scientific, Waltham, MA, USA) (1 abs = 1 mg/mL). All samples were normalized to the same total protein concentration and 50 μL of the sample was added to a 96-well microtiter plate in triplicate. The ELISA assay was performed following the manufacturer’s protocol on three to eight biological replicates run in triplicate (technical replicates). Absorbance readings were measured at 414 nm (SmartReader MR9600; Accuris Instruments, Edison, NJ, USA) and were inversely related to the concentration of estradiol in the samples. Foldchange was calculated based on the value of the control according to the absorbance readings.

### 4.4. Dopamine Concentrations in Whole Eleuthero Embryos and Adult Brains

Zebrafish exposed to atrazine during embryogenesis were collected and processed as described previously at the end of the exposure period (72 hpf) or adult brains (2 ypf) were collected in 200 µL of phosphate buffer solution (PBS), specific to the dopamine ELISA kit protocol. The timepoint 72 hpf was chosen for analysis based on it being the end of embryogenesis and the exposure period. In contrast, 2 ypf was chosen to represent significantly aged fish for comparison. Freeze–thaw cycles, centrifugation, and measurement of the total protein concentration of the supernatant occurred as described previously [64]. All samples were normalized to the same total protein concentration, 50 µL of the sample was added to a 96-well microtiter plate in triplicate, and an ELISA assay was performed following the manufacturer’s protocol [Fish dopamine (DA) ELISA kit (CSB-EQ027496FI); Cusabio, Houston, TX, USA] on four to six biological replicates run in triplicate (technical replicates). Dopamine concentrations were calculated using a standard curve with a four-parameter logistic (4PL) fit in GraphPad Prism (version 10, Boston, MA, USA).

### 4.5. Gene Expression Analysis for Embryonic (1–72 hpf) or Larval (72–120 hpf) Atrazine Exposures

Quantitative polymerase chain reaction (qPCR) was performed on gene targets associated with estrogen and dopamine and the neuroendocrine pathways (i.e., kisspeptin, hypothalamus, pituitary, and dopamine) they are involved in following MIQE guidelines [65]. In total, 24 gene targets (Table 1) were analyzed with the embryonic (1–72 hpf) exposure period, including 8 hypothalamic targets (*avp*, *crhb*, *ghrh*, *gnrh*, *oxt*, *sst1*, *sst3*, *trh*), 6 pituitary targets (*fshb*, *gh1*, *lhb*, *pomca*, *prl*, *tsh*), 6 dopaminergic targets (*drd1a*, *drd1b*, *drd2a*, *drd2b*, *drd2L*, *slc18a2*), and 4 kisspeptin targets (*kiss1*, *kiss2*, *kiss1ra*, *kiss1rb*). A subset of gene targets (*gnrh*, *lhb*, *fshb*, *gh1*, *kiss1*, *kiss2*, *kiss1ra*, *kiss1rb*) was further analyzed with larval-only (72–120 hpf) exposure based on changes observed during the first timepoint as well as significance along the hypothalamus–pituitary–gonadal (HPG) axis. Pools of 45 larvae were rinsed and collected at the end of the exposure period (72 or 120 hpf) and homogenized in 1 mL of TRIzol^®^ Reagent (Thermo Fisher Scientific, Waltham, MA, USA). RNA extraction and clean-up were conducted using the protocol described in [66]. cDNA was synthesized following the manufacturer’s protocol (SuperScript™ IV First-Strand Synthesis System with ezDNase™ Enzyme; Invitrogen, Waltham, MA, USA) and isolated and precipitated using the protocol described in [66]. Primers were designed using the Primer3 website (Table 1). qPCR analysis was performed on a CFX96 Touch Real-Time PCR Detection System (Bio-Rad, Hercules, CA, USA) using SsoAdvanced Universal SYBR^®^ Green Supermix (Bio-Rad, Hercules, CA, USA). The cycling parameters included 3 min incubation at 95 °C and 40 cycles at 95 °C for 15 sec and at 60 °C for 30 sec, followed by a melting curve analysis from 65 °C to 95 °C. Efficiency and specificity were checked by analyzing melt curves, dilution curves, and no-template controls. Gene expression of three to six biological replicates was analyzed in triplicate (technical replicates) and individual gene expression was normalized to beta-actin due to its consistency and lack of alteration by atrazine exposure, as previously reported [23,67,68,69].

### 4.6. Statistical Analysis

Statistically significant changes were tested using a one-way analysis of variance (ANOVA) with SAS software (version 9.4, SAS Institute Inc., Cary, NC, USA) for the detection of alterations in neurohormone concentrations (ELISA) and gene expression (qPCR). A Fisher’s least significant difference (LSD) post-hoc analysis at α = 0.05 was performed following significant ANOVA. All data were confirmed to meet the ANOVA requirements prior to analysis.

## 5. Conclusions

This study used developmentally exposed zebrafish to investigate the effects that atrazine has on hormone and neurotransmitter concentrations immediately following embryogenesis and neurogenesis as well as later in adulthood followed by an exploration of gene targets that could explain these adverse outcomes. This study examined two pathways simultaneously and considered estrogen and dopamine as a neurohormone in the brain, which is a novel approach that can provide insight into a more clearly defined mechanism of atrazine toxicity. The conservation of the neuroendocrine system in zebrafish as well as it being a well-established model to study neurotoxicity can provide support that the perturbations detected following atrazine exposure during development can be translated to humans and be a cause for public health concern. Significant decreases in estradiol concentrations were observed in 0.5 ypf embryonically exposed adult female brains, while an increase was observed in 2.5 ypf zebrafish. Significant decreases in dopamine were detected in 72 hpf atrazine-exposed larvae, while increases occurred in 2 ypf embryonically exposed female and male adult brains. Associated gene expression changes were recognized in *fshb* and *gh1*, with significant decreases in gene expression following 1–72 hpf atrazine exposure and significant increases after both 1–72 and 72–120 hpf exposures. The findings from this study demonstrate the effects that low-dose developmental exposures of atrazine can have immediately as well as delayed and persistent effects. This study only examined one reproductive neuroendocrine hormone and one neurotransmitter, which are altered following atrazine exposure. It is important to investigate other downstream targets based on the gene expression analysis, which found significant changes in *fshb*, another common neuroendocrine hormone. It is also important to follow up with an examination of functional changes to provide further support for the significant changes in dopamine observed in this study. The kisspeptin system should also be more closely examined at timepoints where the system is fully developed to allow for the detection of any changes that may be a result of atrazine exposure during development. Further studies are needed to elucidate a central target of atrazine toxicity that connects the multitude of adverse effects that have been and are continuously being reported in the literature.

## Figures and Tables

**Table 1:** Gene targets associated with hypothalamic, pituitary, kisspeptin, and dopamine systems.

Gene Name	Gene Symbol	Primer Sequences	NCBI RefSeq	Function
Hypothalamic Targets				
Arginine Vasopressin	*avp*	CTGTCTGTGTGTGTGCTGTG GGATCTCTTGCCTCCTCGTG	NM_178293.2	Makes vasopressin
Corticotropin-Releasing Hormone b	*crhb*	TACGAGAAGTACTGGAGATGGC TGATGGAAAAGCAGCACTATGG	NM_001007379.1	Regulates HPA axis
Growth Hormone-Releasing Hormone	*ghrh*	AGGCTGTATTTTGCATCCGT TGGCATCATTTCAAAGCAGGAG	NM_001080092.1	Triggers growth Hormone release
Gonadotropin-Releasing Hormone	*gnrh*	CAGTGCTGTCTATTCCTGCTGA CCCCGTCTGTCTGGAAATCTTT	NM_182887.2	Triggers LH and FSH synthesis and release
Oxytocin	*oxt*	CTACATCTCAAACTGCCCCATC CACACGGAGAAGGGAGAAAATC	NM_178291.2	Plays a role in childbirth and behavior
Somatostatin 1	*sst1*	CAGATACACACTCGCAGACCTG TCCAGACGCACATCATCTTTCT	NM_183070.1	Regulates the endocrine system
Somatostatin 3	*sst3*	GCCGCACTTTAACTTCAACCAT TACTTTGTGCTGGGTTCCTCTC	NM_001045431.2	Regulates sleep and locomotor activity
Thyrotropin-Releasing Hormone	*trh*	GGAAACACACCTGTCTCTCCAT TTGAGGAGACAGTCTGAACGTG	NM_001012365.2	Triggers thyrotropin release
Pituitary Targets				
Follicle-Stimulating Hormone b	*fshb*	TGATTCAGTCTTCGTGTACCCC AGTGCTCTAGTGTATGCTGCAG	NM_205624.1	Develops reproductive organs, regulates the function of sperm, modifies androgens into estradiol
Growth Hormone 1	*gh1*	CTTCGTATCTCTTTCCGCCTCA CGCCAGTTTCTCAGTGATTTGG	NM_001020492.2	Regulates the growth of tissues
Luteinizing Hormone	*lhb*	GTGCACCATAAACACTTCCGAC CTCGACTGTGTGTGTAGGTTGA	NM_205622.2	Triggers the production of testosterone in males and estrogen and progesterone in females
Adenocorticotropin Hormone	*pomca*	TTTCCTACCTGCACAACCCATT GGATGTGTCTGGTTGTCTTTGC	NM_181438.3	Regulates cortisol and androgen production
Prolactin	*prl*	AGAACGCAACACCATTAACAGC CCATTAAACGGGAGAGTGGACA	NM_181437.3	Regulates lactation and breast development
Thyroid-Stimulating Hormone	*tsh*	GCCACCTATCATGTCTCTCCTG CAGCCACACAGTAATTGCACTC	NM_181494.2	Stimulates thyroid hormone production
Kisspeptin Targets				
Kisspeptin 1	*kiss1*	TCTAAACTCTCAGCGCTCTTCT TGTCCTGTTCTCTCTTGCCATA	NM_001113489.1	Regulates dopamine release
Kisspeptin 2	*kiss2*	CGACTCTGACAGACTCAAACAC AGAAAATCGCATCCTTCTGACG	NM_001142585.1	Regulates reproduction and GnRH surge
Kisspeptin 1 Receptor a	*kiss1ra*	CCTAACTTCAAGGCCAACTACG TGTGTCTGAAGAGGAAGGGAAA	NM_001105679.2	Kisspeptin receptor with affinity for Kisspeptin 1 and Kisspeptin 2
Kisspeptin 1 Receptor b	*kiss1rb*	ATCTTGCCACCACCGATATACT TGACCAGGCGACACATAAAATC	NM_001110531.1	Kisspeptin receptor with affinity for Kisspeptin 1
Dopamine Targets				
Dopamine Receptor D1a	*drd1a*	CTTTTGGGATGCCAGAGACTCT TTTCCGTCATTTTCCAACAGCC	XM_021481157.1	Dopamine receptor
Dopamine Receptor D1b	*drd1b*	ATCTGCGCTCTAAAGTCACCAA TGACGCACAGATTCAAGATGGA	NM_001135976.2	Dopamine receptor
Dopamine Receptor D2a	*drd2a*	ACTGACATATCACCTCCATCGC GCTAACATCTGAGTGGCCTTCT	NM_183068.1	Dopamine receptor
Dopamine Receptor D2b	*drd2b*	ATGGCTCTGTGTGTGAAATTGC GTTTAGTGTTGACCCGTTTCCG	NM_197936.1	Dopamine receptor
Dopamine Receptor D2 like	*drd2L*	TCCGCCGTATAACTTCTATGCC TGAGGTAATTGGTGGTGGTCTG	NM_197935.1	Dopamine receptor
Danio rerio Solute Carrier Family 18 Member 2	*slc18a2*	TCGGTGTATGGAAGTGTGTACG AGGAGCAAACATGATGTCCACT	NM_001256225.2	Plays a role in monoaminergic neurotransmission

## Data Availability

The original contributions presented in this study are included in the article/Supplementary Material. Further inquiries can be directed to the corresponding author.

## Supplementary Materials

The following supporting information can be downloaded at https://www.mdpi.com/article/10.3390/ijms252313066/s1.
