# Supplementary material for: Integrated Analysis of Neuroendocrine and Neurotransmission Pathways Following Developmental Atrazine Exposure in Zebrafish
Source: Int J Mol Sci. 2024 Dec 5;25(23):13066. doi: 10.3390/ijms252313066 (PMC11641385; doi:10.3390/ijms252313066)
Supplement: Supplementary file 1 [file ijms-25-13066-s001.zip › ijms-3320587-supplementary.pdf]

SUPPLEMENTARY MATERIAL

Integrated analysis of neuroendocrine and neurotransmission pathways following developmental atrazine exposure in zebrafish

Sydney C. Stradtman, Jenna N. Swihart, Kaylin Moore, Isabelle N. Akoro, Janiel K Ahkin Chin Tai, Wagner Antonio Tamagno, and Jennifer L. Freeman

School of Health Sciences, Purdue University, West Lafayette, IN, USA

Supplemental Figure S1: Methods of dosing and collection of developing whole zebrafish and adult zebrafish brains for ELISA analysis as well as dosing and collection of zebrafish embryos and larvae for qPCR analysis.

Supplementary Figure S2: Measurement of estradiol concentrations in whole 72 hpf fish and adult male brains.

Supplementary Figure S3: Hypothalamic targets were analyzed for changes in gene expression following an embryonic atrazine exposure (1-72 hpf) to 0, 0.3, 3, or 30 ppb at 72 hpf.

Supplementary Figure S4: Pituitary targets were analyzed for changes in gene expression following an embryonic atrazine exposure (1-72 hpf) to 0, 0.3, 3, or 30 ppb at 72 hpf.

Supplementary Figure 5S: Dopaminergic targets were analyzed for changes in gene expression following an embryonic atrazine exposure (1-72 hpf) to 0, 0.3, 3, or 30 ppb at 72 hpf.

Supplementary Figure S6: Kisspeptin targets were analyzed for changes in gene expression following an embryonic atrazine exposure (1-72 hpf) or larval atrazine exposure (72-120 hpf) to 0, 0.3, 3, or 30 ppb and immediately analyzed at end of exposure period (72 or 120 hpf).

Supplementary Figure S7: Expression analysis of select gene targets associated with the hypothalamus or pituitary following a larval atrazine exposure (72-120 hpf) to 0, 0.3, 3, or 30 ppb at 120 hpf.

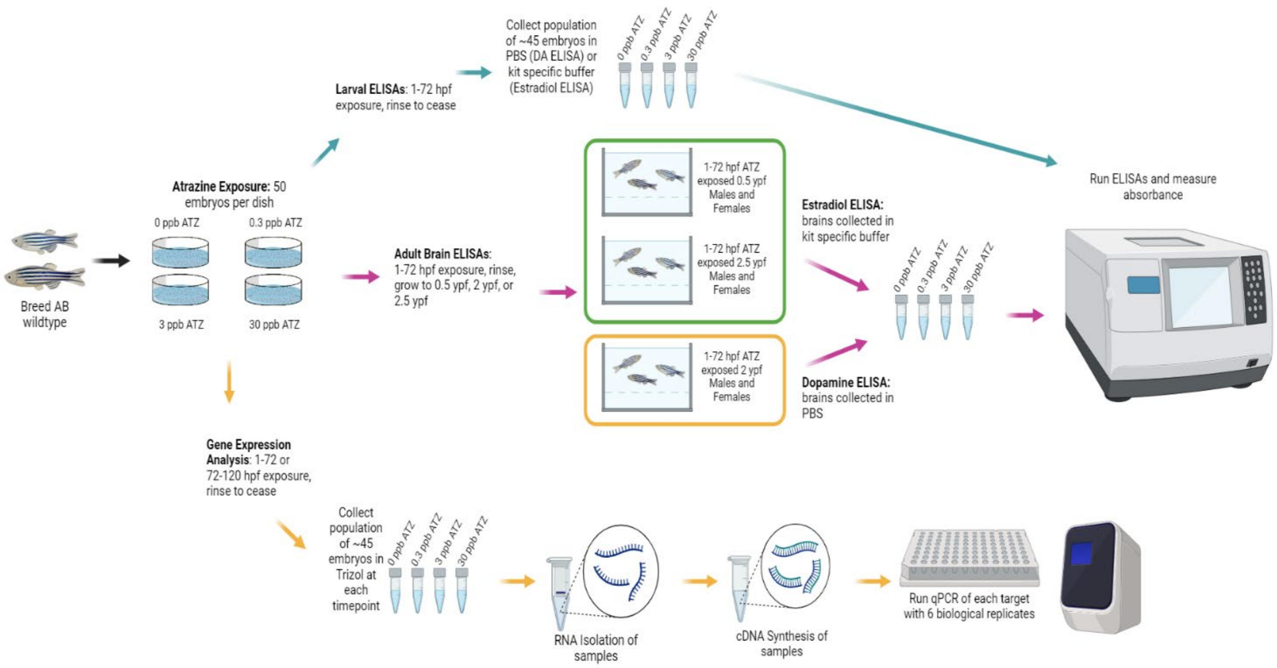

Supplemental Figure S1: Methods of dosing and collection of developing whole zebrafish and adult zebrafish brains for ELISA analysis as well as dosing and collection of zebrafish embryos and larvae for qPCR analysis. AB wildtype zebrafish embryos were collected at 1 hpf and placed randomly in groups of 50 in a Petri dish with either a 0, 0.3, 3, or 30 ppb treatment. In exposure period 1, atrazine treatment was throughout embryogenesis (1-72 hpf). At 72 hpf, exposure was ceased and eleuthero-embryos rinsed. Fish were either collected immediately at 72 hpf for estradiol or dopamine ELISA or gene expression analysis or placed in filtered fish water only and grown to 0.5 years post fertilization (ypf), 2 years post fertilization (ypf), or 2.5 ypf for sex-specific estradiol or dopamine ELISA analysis of the brain. In the exposure period 2, after collection at 1 hpf, groups of 50 embryos were placed in Petri dishes in filtered fish water only until 72 hpf. Fish were then exposed to one of the four atrazine treatments from 72-120 hpf. At 120 hpf, atrazine exposure was ceased, larvae rinsed, and prepared for gene expression analysis.

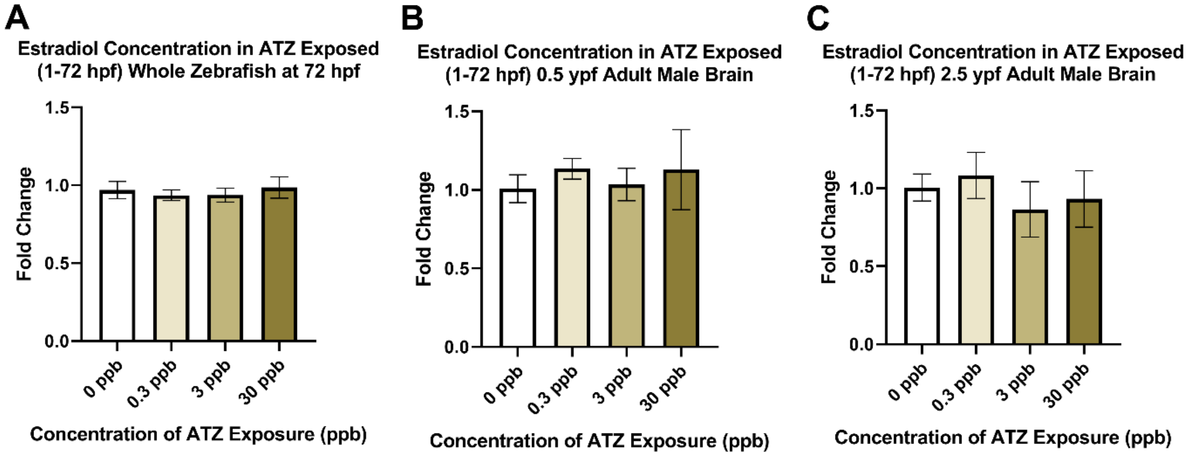

Supplementary Figure S2: Measurement of estradiol concentrations in whole 72 hpf fish and adult male brains. Estradiol concentration was measured after 0, 0.3, 3, or 30 ppb embryonic atrazine exposure (1-72 hpf). Populations of eleuthero-embryos were collected at 72 hpf (A) and adult male brains were collected at 0.5 ypf (B) and 2.5 ypf (C). No significant changes in estradiol were observed at 72 hpf whole fish ($p>0.05$, n= 6), 0.5 ypf adult male brains ($p>0.05$, n=6), or 2.5 ypf adult male brains ($p>0.05$, n=6). Error bars indicate standard deviation.

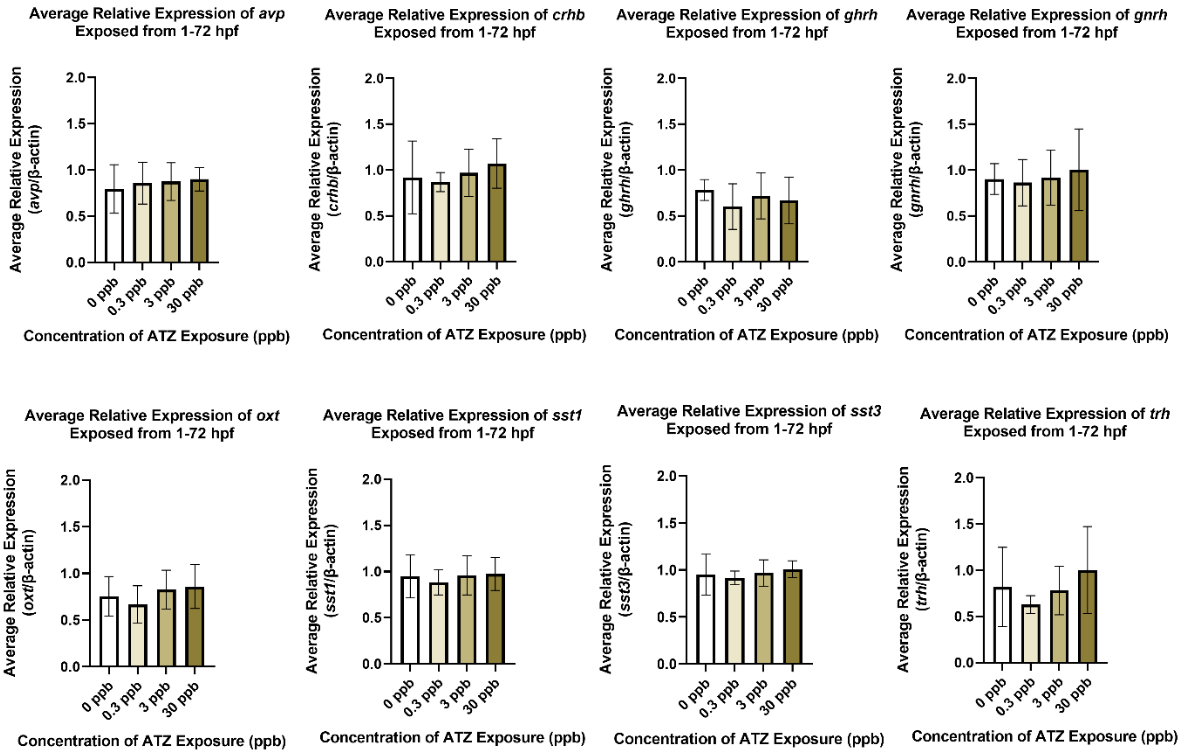

Supplementary Figure S3: Hypothalamic targets were analyzed for changes in gene expression following an embryonic atrazine exposure (1-72 hpf) to 0, 0.3, 3, or 30 ppb at 72 hpf. No significant changes in gene expression were detected in *avp* (A) ($p>0.05$, n=6), *crhb* (B) ($p>0.05$, n=6), *ghrh* (C) ($p>0.05$, n=6), *gnrh* (D) ($p>0.05$, n=6), *oxt* (E) ($p>0.05$, n=6), *sst1* (F) ($p>0.05$, n=6), *sst3* (F) ($p>0.05$, n=6), or *trh* (G) ($p>0.05$, n=6). Error bars indicate standard deviation.

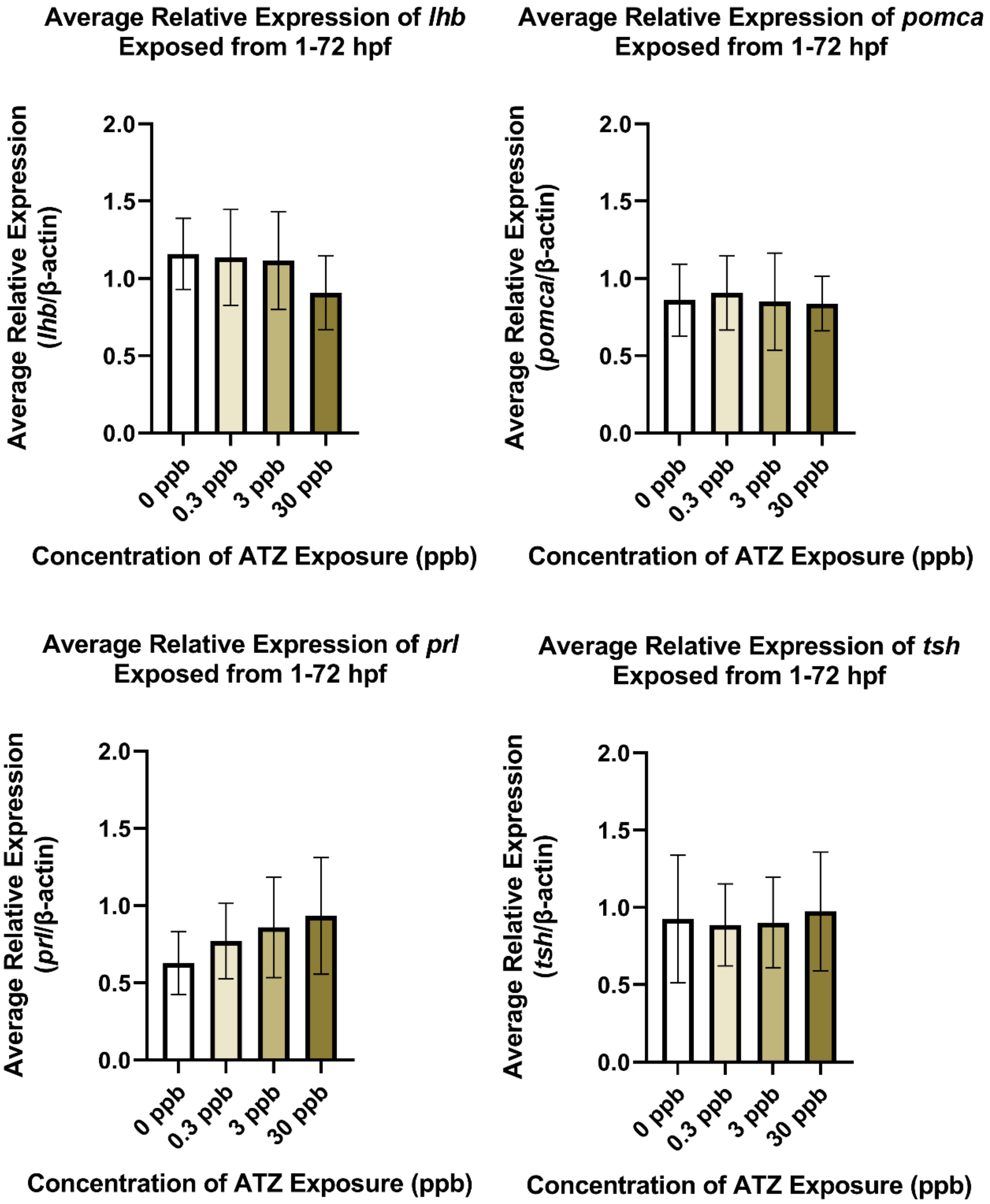

Supplementary Figure S4: Pituitary targets were analyzed for changes in gene expression following an embryonic atrazine exposure (1-72 hpf) to 0, 0.3, 3, or 30 ppb at 72 hpf. No significant changes in gene expression were detected in *lhb* (A) ($p>0.05$, n=5), *pomca* (B) ($p>0.05$, n=5), *prl* (C) ($p>0.05$, n=6), or *tsh* (D) ($p>0.05$, n=6). Error bars indicate standard deviation.

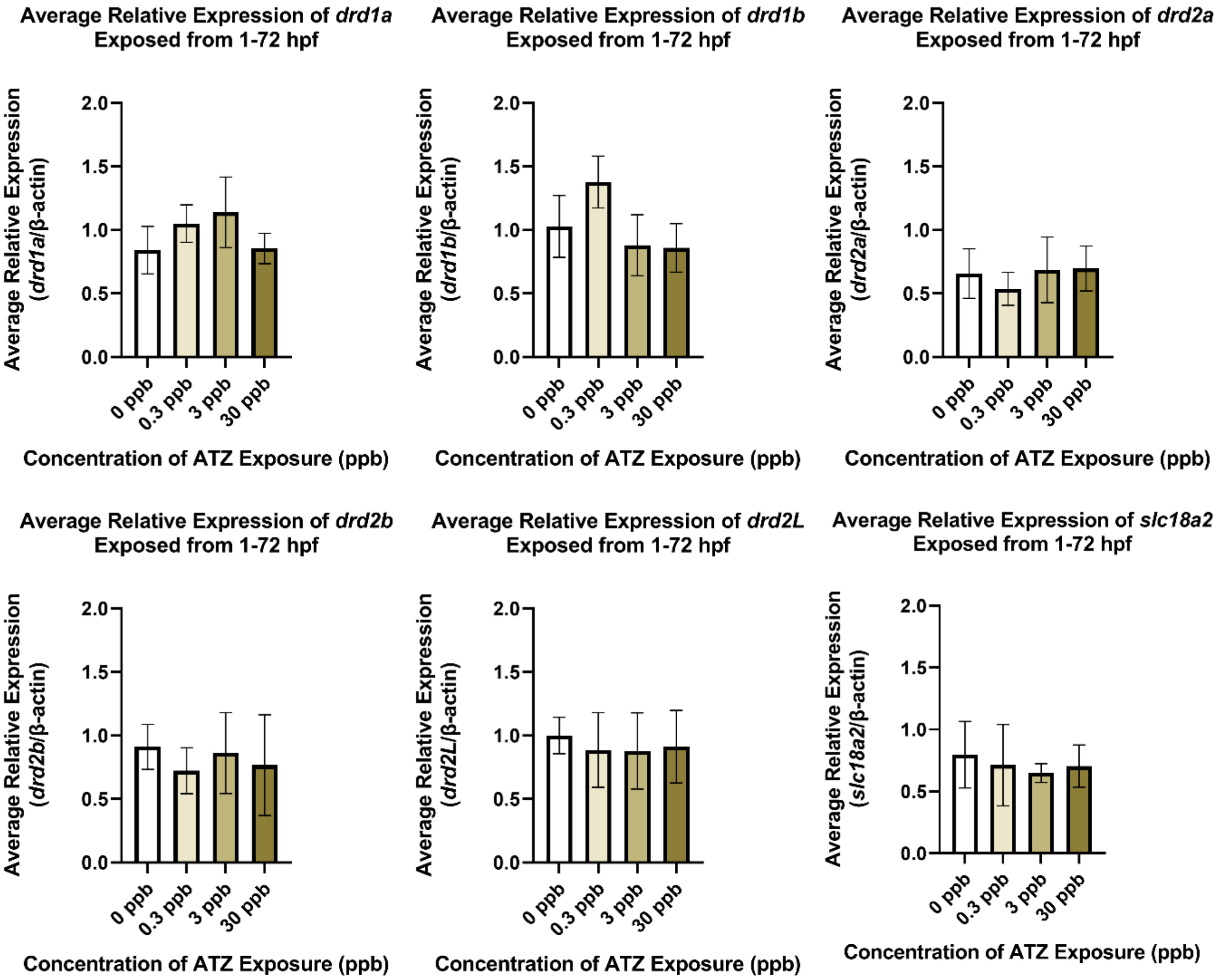

Supplementary Figure S5: Dopaminergic targets were analyzed for changes in gene expression following an embryonic atrazine exposure (1-72 hpf) to 0, 0.3, 3, or 30 ppb at 72 hpf. No significant changes in gene expression were detected in *drd1a* (A) ($p>0.05$, $n=4$), *drd1b* (B) ($p>0.05$, $n=3$), *drd2a* (C) ($p>0.05$, $n=6$), *drd2b* (D) ($p>0.05$, $n=4$), *drd2L* (E) ($p>0.05$, $n=6$), or *slc18a2* (F) ($p>0.05$, $n=4$). Error bars indicate standard deviation.

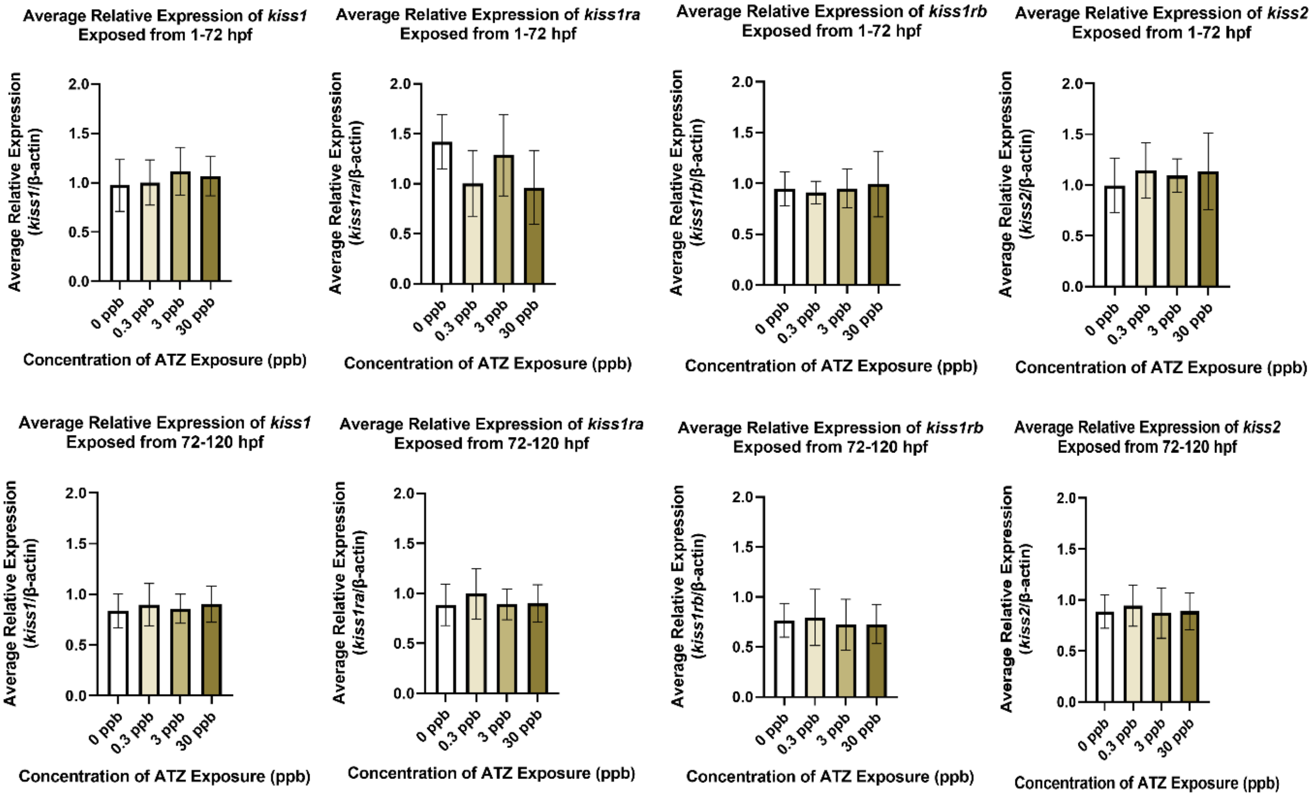

Supplementary Figure S6: Kisspeptin targets were analyzed for changes in gene expression following an embryonic atrazine exposure (1-72 hpf) or larval atrazine exposure (72-120 hpf) to 0, 0.3, 3, or 30 ppb and immediately analyzed at end of exposure period (72 or 120 hpf). No significant changes in gene expression were detected at either timepoint in *kiss1* (A, E) ($p>0.05$, n=5-9), *kiss1ra* (B, F) ($p>0.05$, n=6), *kiss1rb* (C, G) ($p>0.05$, n=5-8), or *kiss2* (D, H) ($p>0.05$, n=6). Error bars indicate standard deviation.

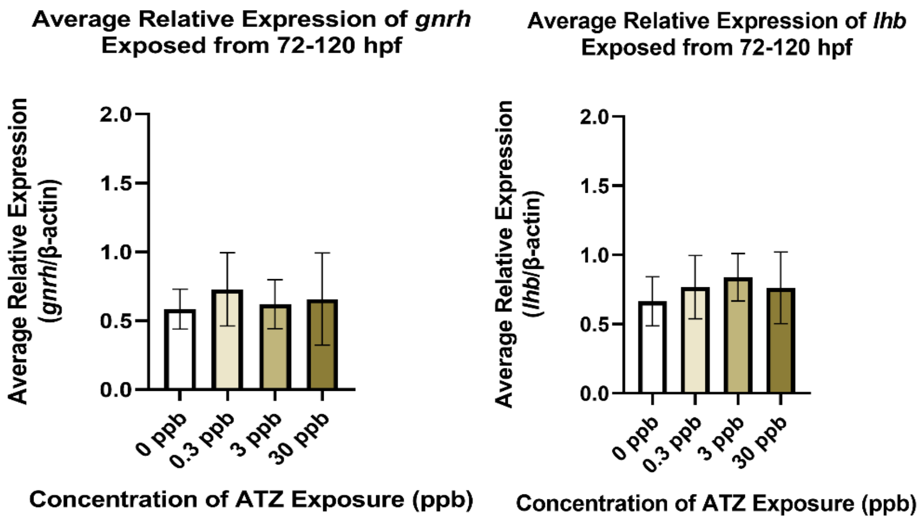

Supplementary Figure S7: Expression analysis of select gene targets associated with the hypothalamus or pituitary following a larval atrazine exposure (72-120 hpf) to 0, 0.3, 3, or 30 ppb at 120 hpf. No significant changes in gene expression were detected in *gnrh* (A) ($p>0.05$, n=6) or *lhb* (B) ($p>0.05$, n=6). Error bars indicate standard deviation.
